# Quantitative Analysis of Food Safety Policy—Based on Text Mining Methods

**DOI:** 10.3390/foods11213421

**Published:** 2022-10-28

**Authors:** Cen Song, Jiaming Guo, Fatemeh Gholizadeh, Jun Zhuang

**Affiliations:** 1 School of Economics and Management, China University of Petroleum, Beijing 102249, China; 2 Department of Industrial and Systems Engineering, University at Buffalo, Buffalo, NY 14260, USA

**Keywords:** food safety policy, central and local government, text mining, cluster analysis, LDA

## Abstract

Recently, food safety and cold chain food have been closely related to the epidemic. The party and the state have intensified efforts to solve food safety problems and prevent possible epidemic risks. China has issued a series of policies and plans to strengthen food safety supervision to improve the food safety policy system. To our knowledge, little work has studied policy problems of food safety with in-depth quantitative analysis for an extended period. In accordance with the different national policies and regulations for food safety, this paper fills the gap by analyzing the policies and comparing the central and local policies issued in China from 2007–2022. In addition, the Latent Dirichlet Allocation (LDA) topic model and K-Means clustering model are constructed to analyze the content of food safety policies and identify hot topics. Finally, a quantitative analysis of China’s food safety policies is made from four aspects: the number of policy release years, the distribution area, the range of action, and the affiliated institutions. The results show that: (a) there is a partial surge in food safety policies issued in 2007, 2011, and 2017; (b) the local food safety policy has a high inheritance to the central policy content, and the trends of annual publication number are highly consistent; (c) the innovation of different policy contents in the region have their own characteristics; (d) the proportion of compulsory and capacity policies is much more significant than that of other types of policies. This paper provides some novel insights into food safety policies.

## 1. Introduction

Although the food safety situation in China continues to improve, there are still some difficulties and challenges in food safety supervision. In 2021, the Beijing Municipal and District Market Supervision Bureau carried out special food safety supervision and spot check work for catering enterprises such as viral food restaurants, chain restaurants, and food courts (BeijingDaily, 2021) [1]. As the most effective method for regulating food safety issues, the central and local governments have issued a series of policy measures to strengthen food safety supervision.

On 28 September 2021, the International Conference on Food Safety and Health, co-organized by the Chinese Society for Food Science and Technology and the International Union of Food Science and Technology, was held in Beijing (Xinhuanet, 2022) [2]. The conference’s topic was “New Needs and Challenges for Food Safety and Health in the Post-Epidemic Era”, which addressed the latest issues in the food sector at home and abroad. The White Paper on Food Safety Best Practices focuses on four main topics and includes best practices provided by leading companies, which demonstrates that China is paying more and more attention to food safety management. Based on the relevant policies of the food industry in China from 2007 to 2022, it can be found that the policies issued by the central government concentrate on strengthening supervision and unifying norms of the industry. Each city actively implements the national development strategy in terms of local policies. According to the actual situation and existing local development problems, relevant policies and measures are formulated to improve the access threshold of the food industry, avoid inferior products disturbing the market order, and promote the healthy development of the food industry.

Text mining enables people to quickly discover useful information from a large amount of redundant information and reveal the connections between various information. Based on the text mining approach, this paper analyzes and compares the characteristics of central and local food safety policy development, explores the shortcomings of the existing food safety policy system, and makes suggestions to improve the food safety policy system.

## 2. Literature Review

### 2.1. Text Mining on Policies and Topic Model

As an emerging field in text mining, policy text mining has been studied by an increasing number of scholars in this area in recent years. Han et al. (2019) used text mining, potential semantic analysis, and other technologies to extract policy elements with the relevant policies of state-owned capital layout and reorganization. The non-interventionist approach avoids the validity flaws caused by the subjective bias of researchers and provides more objective policy recommendations for policymakers from a neutral perspective [3]. Miao et al. (2021) used text mining technology to construct a policy evaluation model using logistics policies from three Chinese provinces. They scored the logistics policy texts using the Policy Modeling Consistency (PMC) model to visually reflect the focus of logistics policy documents in different provinces [4]. Park et al. (2022) analyzed 103,428 Google Maps reviews from major U.S. hub airports to identify representative topics of passenger concern and to further investigate spatial drivers and policy compliance in an epidemic environment [5]. Gao et al. (2021) combined text mining and machine learning methods to propose a modular policy evaluation system, which includes data acquisition, data processing, index evaluation construction, and score evaluation. Compared with the traditional policy evaluation method, it has higher accuracy, objectivity, and efficiency, which is helpful for the government’s policy implementation [6].

The LDA model, as a topic model for semantic extraction of text information, is widely used in text classification, text clustering, abstract extraction, and sentiment analysis (Zhu et al. 2017) [7]. Jiang et al. (2012) used the CUDA toolkit provided by NVDIA to optimize the traditional LDA program, which significantly accelerated the program’s speed when processing documents [8]. Jeon and Kim (2015) proposed a spatial class LDA model for image classification, which significantly improves image classification accuracy [9]. Wei (2017) fused LDA with fuzzy k-nearest neighbors and proposed a fully fuzzy LDA method to enhance the recognition performance of the model [10]. Shi et al. (2017) proposed an augmented LDA model using high-quality word vectors. As a result, the cumulative accuracy of the model under various metrics increased by 5.3% on average [11]. In addition, text clustering is an important research direction in the field of text mining. It has important application value in the organization and browsing of large-scale text sets and the automatic generation of text set classification (Shi and Han 2010) [12]. As one of the classical clustering algorithms based on partition, the K-means algorithm is widely used in text clustering (Yuan et al. 2019) [13]. Li et al. (2017) proposed a distributed improved K-means algorithm based on Hadoop, which overcomes the problem that traditional algorithms are easy to fall into local optimal solution due to the uncertainty of the initial center point [14]. Yang et al. (2016) proposed an improved K-means clustering algorithm based on Adaptive Cuckoo Search, which improves the search accuracy and convergence speed [15].

### 2.2. Research on Food Safety

#### 2.2.1. Related Technologies Used in the Field of Food

The use of modern scientific techniques to ensure food safety and guarantee the quality of food and drugs has attracted the attention of many researchers. Wu and Sun (2013) found that hyperspectral imaging is a non-destructive and rapid method for food quality and safety analysis and assessment [16]. Bai and Liu (2015) introduced the applications of nanotechnology and nanomaterials in food processing, food packaging, food machinery, food testing, and food traceability [17]. Jespersen et al. (2016) measured the food safety culture in the food manufacturing industry through the food safety maturity model to determine the focus of strengthening the food safety culture [18]. Nyarugwe et al. (2020) believed that understanding national values and food safety governance methods would influence food safety culture in different ways, which is expected to develop the best methods for companies operating to improve food safety performance [19]. Fan et al. (2020) studied the progress of carbon precursor utilization of food waste and its application in food safety testing and concluded that food waste has the potential to prepare carbon quantum dots (CQDs) [20]. Tao et al. (2019) proposed a hierarchical multi-domain blockchain (HMDBC) network structure and secondary inspection mechanism to improve the traditional food regulatory system with problems such as a lack of industry chain and data fragmentation [21]. Thangalakshmi et al. (2021) introduced 3D food printing technology and experimented with the best proportional composition of raw materials for 3D printability [22].

#### 2.2.2. Research on Food Logistics

Food logistics have been widely studied, including controlling food risks in the supply chain and the food early warning system. Ng and Yang (2009) proposed using the mass media’s public wisdom to improve the existing food supply chain framework [23]. Zhang (2014) proposed a framework for a food safety early warning system based on consumer feedback, which can effectively avoid the outbreak of widespread food safety events and reduce food safety risks in China [24]. Zheng et al. (2021) proposed a food safety traceability system based on RFID two-dimensional code technology and big data storage technology, which can trace the whole process of food production information and is conducive to epidemic prevention and control [25]. Henrichs (2021) used an adaptive system in the food supply chain to reduce food waste and improve food safety [26]. Anand and Saxena (2022) believed that food safety and quality issues are critical during the COVID-19 pandemic and explored the application of IOT devices for safe-packaged food and frozen food to improve food standards [27].

#### 2.2.3. Food Safety Policy

There is a stream of research on food safety-related policies. Basha (2014) suggested that policymakers should develop appropriate marketing strategies to promote organic food as a healthier and safer food for society [28]. Simone et al. (2019) explored whether food safety policies affect the support, risk control, and time preferences of respondents and found that good news and bad news affect preferences and welfare measures [29]. Li et al. (2021) systematically analyzed the food safety-related literature and policies and identified the factors influencing food safety problems in China. They divide the evolution of China’s pollution-based food safety policies into four stages [30]. However, most scholars study food safety policies from a qualitative perspective, and few conduct quantitative analysis. Ni (2017) used the quantitative method of policy text to study the structure and characteristics of China’s food safety policy under the current regulatory system [31].

In all, most of the research on food safety is on the technical aspects and risk control. However, few articles systematically review food safety policies and analyze the policies’ role in the regulatory system. In addition, few papers on food safety policy grooming suffer from a possible lack of comprehensiveness in selecting, collecting, and organizing policy texts. Based on the LDA topic model and K-Means clustering model, this paper analyzed the food safety policies issued by the central and local governments, respectively, from a quantitative perspective that included the number of policy release years, the distribution area, the range of action, and the affiliated institutions.

**Figure 1:**
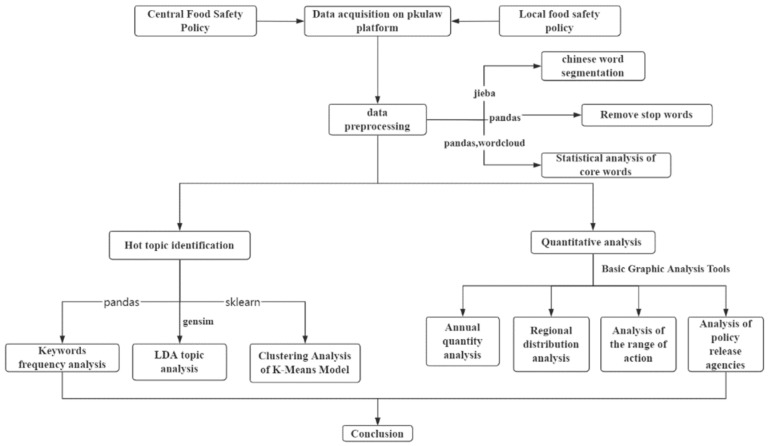
The methodological approach followed in this study.

The remainder of this paper is structured as follows: Section 3 introduces operations such as data acquisition and preprocessing and develops a preliminary understanding of the topics of policy texts. Section 4 uses the LDA and K-Means model for topic recognition of policy texts, and the results are compared and analyzed. Section 5 quantifies food safety policies from four perspectives and provides a specific analysis. Finally, Section 6 summarizes the research results. The flowchart in Figure 1 shows the methodology that we have followed in this research.

## 3. Data Processing and Statistical Analysis of Core Words

### 3.1. Data Collection for Food Safety Policy

In the PKULAW dataset, the central and local food safety policies issued in China from 2007 to 2022 are crawled, including title, release department, release date, timeliness, effectiveness level, legal category, and policy-specific content. The vacancy value is eliminated for all of the obtained policy documents, and the duplicate removal operation is carried out. Although only the document number of the central food safety policy is collected, one of the local food safety policies is not collected. Therefore, Python has been mainly been used to de-duplicate documents with the same title. The data in each document are arranged according to the release date of the policy, while a total of 10,180 target data are obtained.

### 3.2. Statistical Analysis of Core Words

After word segmentation and stop words, the effective keywords and the frequency of the target data obtained by the central and local food safety policies are analyzed. The top 20 high-frequency keywords of the central and local food safety policies are: food and drug, agricultural products, additives, catering, health, product quality, safety accidents, school canteens, diet, raw materials, quarantine, safety hazards, family-planning commission, food poisoning, pesticides, disinfection, shelf life, infants, butcher, and dairy products. It has been found that the food safety policies issued by the central government are highly consistent with those issued by the local government, who focus on agricultural products and additives.

Agricultural products are one of the most widely consumed food in China, which require increasingly high quality. Currently, China’s agriculture has fully entered the global economic competition stage, which pays increasing attention to the safety control of agricultural products that are conducive to increasing market competitiveness. As an essential part of the modern food industry, food additives have greatly promoted the development of the food industry. However, controlling the production, procurement, storage, and other aspects is also very important. Irrational use of additives can cause significant harm to the human body. Therefore, both the central and local governments regard the standardization of the production and use of food additives as a focus on food safety. However, the central government policies are mostly macros, such as laws and regulations, guidance, and planning outlines. Policies formulated by local ministries are more relevant to their own practicality, primarily because of announcements and notifications.

## 4. Hot Topic Identification

### 4.1. Keyword Analysis of Food Safety Policies

From 2007 to 2021, the central government issued four policy documents. This paper divides the time between the four policy documents into one stage, so the four documents are separated into three stages. Because the policies issued in 2022 have not yet been fully collected, the number is small; therefore, the policies of 2022 and 2021 are merged. Based on the central release of food safety planning at different times, the three stages of food safety development are determined.

#### 4.1.1. Keywords Frequency Analysis of Central Food Safety Policy

**Figure 2:**
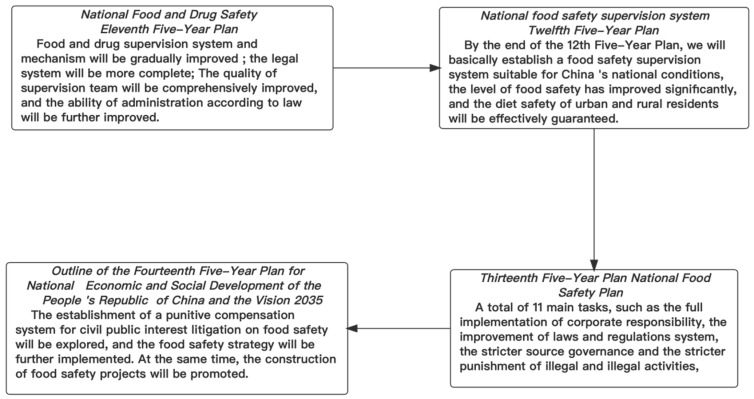
The Policy development process of the food safety industry.

According to China’s food safety policy course, the central food safety policy research is divided into three phases. The first phase is between the “Eleventh Five-Year Plan” and the “Twelfth Five-Year Plan”, which corresponds to the period from 2007 to 2012. The second phase is between the “Twelfth Five-Year Plan” and the “Thirteenth Five-Year Plan”, which corresponds to the period from 2013 to 2017. Finally, the third phase is between the “Thirteenth Five-Year Plan” and the “Fourteenth Five-Year Plan”, which corresponds to the period from 2018 to 2022. The research direction of food safety in these three stages is studied, and the course of China’s food safety policy is shown in Figure 2.

Table 1 shows the central food safety policy’s annual keywords and corresponding word frequency information. There are three main keyword topics for food safety in China: the first is the main upper regulatory authority for food safety, which includes the National Medical Products Administration (NMPA) and the China Entry-Exit Inspection and Quarantine (CIQ); the second is high-risk food safety products, which mainly includes healthcare food, infant milk powder, tableware, and alcohol; finally, the third is the main object of food safety and places such as food markets, canteens, and sellers.

#### 4.1.2. Keywords Frequency Analysis of Local Food Safety Policies

Table 2 shows the local food safety policy’s annual keywords and corresponding word frequency information. Comparing the keywords in the food safety policies issued by the central and local governments shows a high degree of consistency between the policies. This may be because the local government should first formulate policies around the general framework of policies formulated by the central government and then adjust the policies to suit the economic and social development of the region and consider the specific local conditions. The keywords’ frequencies show that local governments are determined to maintain stability and build a strong defense line by focusing on aquatic products, canteens, and food and drug products. At the same time, they comprehensively investigate hidden dangers, strictly control the use of various additives and fake and shoddy products, and deepen special rectification. At the National Conference on Food Safety in the Market Supervision System held on 27 April 2022, the government also deployed the next phase of the “guard the bottom line, investigate hidden dangers, ensure safety” special work to ensure the overall stability of food safety was in accordance with the sound development trend.

### 4.2. LDA Topic Analysis

Table 3 shows that the first three topics are food itself. The government has formulated corresponding legal norms for using raw materials and additives and has carried out publicity and education for various enterprises to ensure food safety from the source. The last three topics are aimed at all aspects of food and drug production, such as logistics, distribution, and upper supervision. In all, it can be seen that the government has put forward corresponding policies for the whole food distribution process to properly regulate the food industry market environment. The characteristics of each Topic combined with the actual development of China’s food safety industry and the implementation of China’s policies are analyzed as follows.

(1) Food additives. In order to strengthen the management of food additives, China has introduced a series of national food safety standards and has established a relatively perfect national food safety standard system for food additives. In the whole system, a total of 600 food additive food safety national standards are formulated, which can meet the industry supervision and demand in China. Furthermore, some of the standards in the system have been advanced, such as the “Food safety national standards Food Additives Gum base and its ingredients”. Similarly, China has improved the legal system, revised the standards system, carried out a series of sampling and risk monitoring, and conducted other means of continuously strengthening the supervision of food additives. All these measures show that China has carried out the omnidirectional management of food additives, which can effectively guarantee food safety.
(2) Source tracing. As an effective means to ensure food safety, traceability has always been highly valued by governments, industry organizations, and enterprises. In 2004, the Shandong Institute of Standardization carried out research on the tracking and traceability of the agricultural products supply chain, established the “food safety traceability system” with Chinese characteristics, realized the traceability management of all aspects of product production and circulation, and recorded the product quality-related information from production to packaging. In addition, the additional information data in the process of product circulation is recorded to ensure full traceability of products from production to sales. Although China has effectively promoted the improvement of the traceability system construction, it is still difficult to implement effective tracking and traceability, control, and recall when food safety problems occur. Combined with the current situation of epidemic prevention and control, China continues to do a good job on imported cold chain food “physical defense” work, which strengthens the inspection and control of food related to the epidemic and resolutely puts an end to food safety problems.
(3) Regulation and early warning. As food safety incidents continue to occur frequently, China puts forward various normative measures, such as the “food safety operation norms of catering services” issued by the State Administration for Market Regulation in 2018. In addition, since food safety is a systemic project that includes all aspects, from cultivation to distribution, different aspects face different problems. For these issues, China has established a food safety early warning mechanism to socially supervise and manage food safety to protect workers’ and consumers’ lives and health. However, scholars have more research on food safety supervision and less research on food safety early warning mechanisms and early warning management.
(4) Food logistics. The Food supply chain in China has problems such as high logistics costs, perishable products, and a low degree of informatization. Zhejiang Province and Beijing have implemented the “Network catering service catering safety management specification” and “Takeaway seal” as local legislation in response to consumers’ concerns about the disconnection between safety and health protection in the last mile of takeaway distribution. As the development of food logistics is inevitable, the operation mechanism of the logistics supply chain needs to be improved, and the upper and lower sources of the production chain should be combined. At the same time, the application of cold chain technology should be promoted, and the operation and management system of the cold chain should be optimized to solve the problems of food diversity and fast demand that are closely related to food logistics, which can improve the competitiveness of China’s food enterprises.
(5) Campus security. Campus food safety issues are closely related to the health of adolescents. However, in recent years, incidents in provinces and cities have occurred from time to time. For example, in 2021, students in middle schools in Henan Province detected excessive Escherichia coli in their lunches. In 2018, an international school in Shanghai provided mildew and expired food to children. Therefore, campus catering food safety should not be ignored. How to encourage students to eat healthy and nutritiously is a big concern. Relevant departments in various regions have continued to carry out campus food safety protection actions against this problem and strictly abide by the bottom line of campus food safety. The market supervision department should further improve supervision efficiency, strictly control the risk of campus food safety, and protect the safety of teachers and students.
(6) Upper supervision. Food safety is related to people’s health and is a major event related to the national economy and people’s livelihood. The “14th Five-Year Plan” proposes to strengthen biosafety protection and improve the level of safety and security of people’s health products and services such as food and drugs. With the goal of being “scientific, unified, authoritative, and efficient”, China has continuously deepened the food safety supervision system reform. From decentralized supervision to unified supervision of food safety, and from food safety supervision to food safety governance, China’s food safety supervision has entered a new stage.

### 4.3. Cluster Analysis of Food Safety Policies

The central policy provides official keywords in the PKULAW database, which refines a document’s core information. The central food safety policy data is replaced with the officially given keywords. When searching for policy information, the Peking University magic database will give official keywords to each policy to summarize the main content of the policy. It is believed that the keywords of the official policy content have a good effect on the analysis of hot spots in the policy. Therefore, the keywords of the official food safety policy issued by the central government are combined with the main content of the food safety policy issued by the local government to form a new data set. K-Means clustering is applied to the new data set to further analyze the hot spots of food safety policy in China.

By constructing the K-Means model, the obtained target data are classified into four categories. The categories related to the Department of health, supervisors, safety control, supervision institutions, rights, and interests are classified into the category of supervision and rights protection. The categories related to counterfeit and inferior, market supply, quarantine, and quality are classified into the category of market regulation. The categories related to catering, ordering, business licenses, and vendors are classified into catering licenses. The categories related to food, drug, and product safety are classified into food safety products.

Table 4 shows that the market regulation policy accounts for 73.91%, which is far greater than the other types of policies. This policy demonstrates that the government pays more attention to the quality and safety of food and improves the food safety levels in China by controlling the production and sales of counterfeit and inferior products, which prevents food safety incidents and ensures the output and quality of food. In addition, with the impact of COVID-19, there has been a significant increase in the correlation between the epidemic, food safety, and cold-chain food. In response to the food safety issues of epidemic prevention and control, the government has also increased the supervision and regulation of imported cold chain food.

Comparing the results of the K-Means model with the ones of the LDA topic model, it is found that there are two more topics in LDA than in K-Means, and the policy division of LDA is more detailed. However, they both show that China’s food safety policy focuses on market regulation and early warning. Especially during the epidemic, both models’ results reflect China’s current regulatory focus on quarantine and supervision of imported cold chain foods. With the current epidemic prevention and control situation, the third-party testing agencies, the use of intelligent Internet and food safety rapid inspection means to prevent food safety problems under normal epidemic prevention and control, and the strict implementation of traceability platform management requirements to ensure the traceability of food raw material procurement is advocated. The “Fourteenth Five-Year Plan” for the development of cold chain logistics released by the state in December 2021 calls for an increase in the construction of cold chain logistics supervision warehouses and vigorously promotes the development of cold chain logistics to guarantee quality and safety, which shows that the results of the two models match the current food safety situation.

## 5. Quantitative Analysis of Food Safety Policies

### 5.1. Annual Quantitative Analysis of Policy Texts

#### 5.1.1. Annual Quantity Analysis of the Central Policy

**Figure 3:**
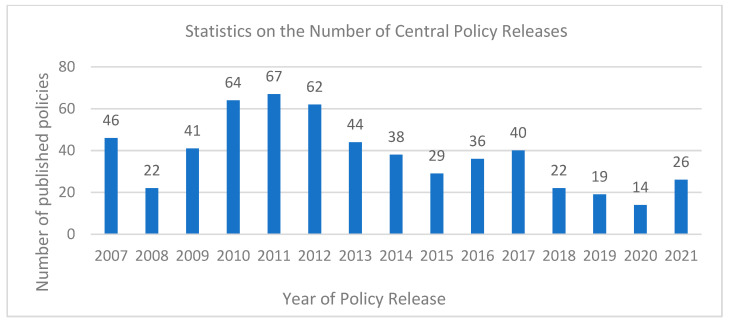
Annual quantitative analysis of the central policy (2007–2021).

The food safety policy can be divided into central policy and local policy. Due to the small number of central policies in 2022 and the difficulty in availability, Figure 3 shows the annual central policies between 2007 and 2021. It is found that the policies issued by the three nodes in 2007, 2011, and 2017 have surged partially, which may be related to the release of the “Eleventh Five-Year Plan”, “Twelfth Five-Year Plan”, and “Thirteen Five-Year Plan”, respectively, in the three years. The number of food safety policies issued by the central authorities in other years has been relatively stable. There are few policies in 2019 and 2020. These policies demonstrate that the task of food safety governance is complicated, the government is very concerned about this, and the attitude is firm to build and improve the food safety governance system with great determination.

#### 5.1.2. Annual Quantity Analysis of the Local Policy

**Figure 4:**
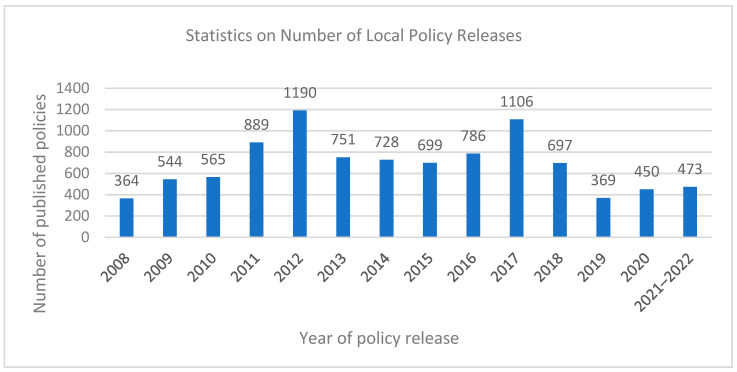
Annual quantitative analysis of the local policy (2008–2022).

From the comparison between Figure 3 and Figure 4, it is found that the number trend of food safety policies issued by the central and local governments is very similar, which indicates that the central and local governments not only maintain a high degree of correlation between policy content, but also ensure a high degree of consistency of policy release time.

### 5.2. Regional Distribution Analysis of Policy Texts

#### 5.2.1. Regional Distribution Analysis of Central Policy

From 2007 to 2021, the central government issued 570 policies, which was significantly less than the local food safety policies. However, the policies issued by the central government are more macro. The central government should ensure enough authority to achieve unified leadership but also make local governments have certain autonomy and creativity so that their enthusiasms are fully played. Specifically, before the 18th National Congress of the Communist Party of China, the food safety strategy is “To rely on the domestic resources and achieve basic self-sufficiency in grain”. After the 18th National Congress of the Communist Party of China, the central government made major adjustments to the strategic policy of food safety, namely “domestic grain production, guaranteed food production capacity, moderate imports, and technological support”, and formed a food security guarantee mechanism.

#### 5.2.2. Regional Distribution Analysis of Local Policy

**Figure 5:**
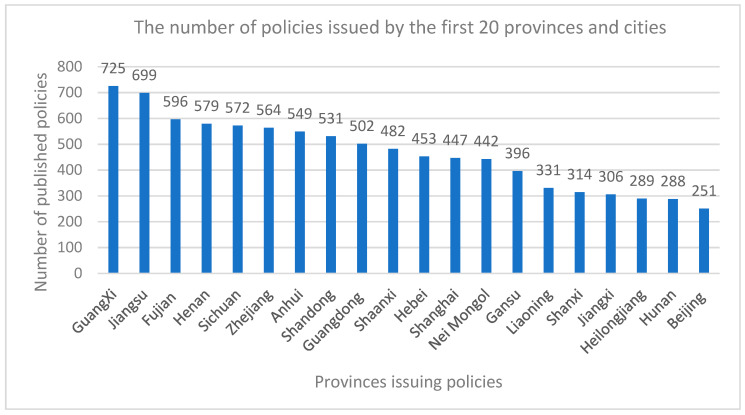
Regional distribution of food safety policies in China (2008–2022).

From 2008 to 2022, the number of policies issued by the local government was 9611, which is very large. Almost all provinces and cities issue local food safety policies. Figure 5 shows the top 20 provinces and cities with the most significant number of policies issued. It is found that China’s food safety policies are unevenly distributed. More policies are issued in the south, especially in Guangxi and Jiangsu Province, which are significantly higher than other provinces and cities, while Beijing has only issued 251, which accounts for approximately 2.6%.

### 5.3. Analysis of the Range of Action of Policy Texts

Although China’s food safety has shown a stable and good situation, the foundation of food safety is still weak, and problems occur from time to time, such as “melamine milk”, “fake mutton”, and “fast-grown chicken”. These problems have illustrated China’s severe food safety situation, and there is still a gap compared with the people’s expectations. Given the aforementioned situation, China has taken a series of measures to ensure food safety: the Food Safety Law was promulgated in 2009, the Food Safety Commission of the State Council was set up in 2010, the China National Center for Food Safety Risk Assessment was established in 2011, the Decision of the State Council on Strengthening Food Safety was issued in 2012, and the National Medical Products Administration was established in 2013. In recent years, the regulatory authorities have successively promulgated laws and regulations such as the Measures for the Administration of Food Production License and Food Operation Approval Administration Measures, as well as industrial policies such as Opinions on Deepening Reform and Strengthening Food Safety Work, as well as Guidance on Promoting Healthy Development of Food Industry. These measures control food safety in China. This subsection will provide a specific classification of the food safety policies issued in China according to the different policy roles, which mainly includes Mandatory, Value, Capability, Awards and Punishments, and Innovation. Since the division of such policies requires careful manual reading of the policy text and the division can only be made after understanding the main content, which is time-consuming, this paper divides the range of action of the 570 policies issued by the central government. It is found that the coverage of China’s food safety policy is comprehensive, and all aspects of the food industry are involved, which promotes the healthy development of China’s food industry.

Table 5 shows the statistics of the number and proportion of the range of action of policy texts. It is found that the market supervision departments are guided by food safety issues, and that they have increased the intensity of spot checks on safety indicators, industries, formats, regions, and related enterprises that have more problems in daily supervision, complaint reporting, law enforcement, punishment and disposal, and risk warning. Specifically, the policy that accounts for the largest proportion is the establishment and implementation of standards in the capability category, which is 16.14%. That is, with the continuous improvement of living standards, people not only pursue food and clothing but also pay more attention to eating safely, healthily, and comfortably. Food safety standards play a vital role in this process. Currently, China’s food safety standards can cover the sales market and the main food categories consumed by people, and the coverage can reach more than 90%. The second is the regulatory system, which accounts for 11.58%. The regulatory system mainly refers to the reform of the food regulatory system, the implementation of superior work, and some comprehensive policies involving multiple dimensions. This part covers a wide range of dimensions. It aims to promote the improvement of the food safety supervision system and comprehensively enhance the ability of food safety supervision, which is of great significance to maintaining people’s health and life safety. The remaining awards and punishments policies, value policies, and innovation policies accounted for less than the mandatory and capability policies, especially the awards and punishments policies, which only accounted for far less than other categories at only 2.11%. This shows that the policy role is unevenly distributed in types. The government should strengthen policy support for these weaknesses, establish positive and negative models and technological innovation, strengthen the comprehensive coordination of food safety, and play a positive role in ensuring public food safety.

### 5.4. Analysis of Policy Release Agencies

The main functions and areas of responsibility of the department are different. The focus of policy formulation is not consistent. For instance, the Ministry of Health is mainly responsible for the formulation of national standards. The Administration of Quality Supervision, Inspection, and Quarantine (AQSIQ) is mainly responsible for the import and export of food, all kinds of food supervision and sampling inspection, and risk monitoring policy formulation. The State Council is mainly responsible for grasping the direction of macro policies. The National Medical Products Administration focuses on strengthening some daily social supervision and spot checks before major festivals and activities.

Table 6 shows the statistics of the release departments of the central food safety policies. Since there are many departments involved in policy releases, this paper merges departments that are not the main release policies into the last line to facilitate readability. This table shows that the National Medical Products Administration (NMPA), the Ministry of Health, the National Health and Family Planning Commission, and the Food Safety Commission of the State Council are the four departments that issue the most policies, accounting for 34.74%, 12.63%, 9.12%, and 6.49%, respectively. Other agencies will also formulate some targeted food safety policies to improve the entire policy system according to the different needs of different regions or industries. For example, according to the problems in the field of pension services, the Ministry of Civil Affairs issued policies requiring the implementation of a food safety management “president responsibility system” and required producers and operators to strictly perform the main responsibility of product safety. The Ministry of Transport issued policies to accelerate the development of cold chain logistics to improve cold chain flow equipment and facilities and encourage the innovative development of cold chain logistics enterprises.

## 6. Conclusions

This paper constructs the LDA and K-Means model to analyze food safety policy texts from 2007 to 2022. The annual number and regional distribution of policies are analyzed from the perspectives of the central and local governments, which emphasizes the types of roles and publishing agencies of central policies. The hot topics of policy release, annual high-frequency keywords and annual change trend, distribution trend, and policy organization distribution are obtained. Our conclusions are as follows:

The focuses of food safety policies issued by the central and local governments and the trends of annual publication numbers are highly consistent. Food safety policy focuses on the supervision of food additives, the construction of a food traceability system, food safety norms and early warning, food logistics, and campus security. According to the trend of food safety development, the focuses of policies are different each year. The peak period of policy release is around 2010–2012, which matches the period of the 11th Five-Year Plan for National Food and Drug Safety issued by the central government. The number of policies issued annually in other years is relatively stable, which demonstrates that the state has a strong attitude towards food safety supervision and that the whole system of food safety supervision is constantly improving.

The local food safety policy has a high inheritance to the central policy content, and the innovation of different regional policy contents have their own characteristics. Specifically, the regional distribution of China’s food safety policies is not balanced, and the policies issued in the south are more than those in the north, especially in the Guangxi, Jiangsu, and Fujian provinces. This disparity may be related to the local economic conditions and the development level of the food safety industry. Similarly, various provinces and cities are actively promoting the development of the local food safety industry in response to the country’s call. Food safety supervision also presents the national, provincial, municipal, and county “four levels” phenomenon, and each level has its concentration. Currently, China’s food safety is guaranteed, but there are still some problems and a certain gap in the expectations of the people. Although food safety issues cannot be “zero risk”, government regulation can be “zero tolerance”.

According to the classification of food safety policies issued by the central government, it can be found that the proportion of mandatory and capability policies is much larger than other types of policies, which shows that the current focus is still on the supervision of sampling and supervision system construction. In the future, the food safety industry should improve continuously strengthening the supervision and sampling of enterprises and units, investigate food safety hazards, prevent and control all kinds of explicit and invisible risks, and carry out special rectification of outstanding problems of food safety. In addition, it needs to ensure the healthy growth of students, focus on the supervision of school food safety and continue to strengthen supervision. Finally, it is necessary to crack down on illegal activities and focus on the governance of products with poor quality in accordance with the law.

The research in this paper can be improved in other ways as follows:

The data acquisition. The raw data can be selected according to the different departments of the release policy by year. We suggest to record the policy’s document number during the retrieval process for subsequent deduplication and text analysis.

The division method of the range of action of the policy. It is better to use a more objective, accurate, and fast division method instead of manual division to ensure that the locally-issued policies, as well as the centrally-issued policies, are divided.

The analysis of policy-affiliated institutions. Besides the analysis of the single policy release department, the joint policy release department should be analyzed and compared.

## Figures and Tables

**Table 1:** Annual keywords of national food safety policy.

2007–2012		2013–2017		2018–2022	
Keywords	Word Frequency	Keywords	Word Frequency	Keywords	Word Frequency
Additive	259	National Food Safety Standards	449	Additive	287
NMPA	216	Additive	315	National Food Safety Standards	205
National Food Safety Standards	191	Food and Drug	311	Infant	59
School Canteen	140	Recipes	122	Expiration Date	57
Product Quality	136	Healthcare Food	122	Tableware	33
Safety Accidents	121	Milk Powder	72	Sellers	29
Healthcare Food	113	Expiration Date	43	Date of Manufacture	27
Food Market	69	Level Measurement of Residue	31	Canteen	25
CIQ	59	Alcohol Products	28	Responsible Person	23
Enforcement Inspection	53	Date of Manufacture	27	Failure Rate	23

**Table 2:** Annual keywords of local food safety policy.

2008–2012		2013–2017		2018–2022	
Keywords	Word Frequency	Keywords	Word Frequency	Keywords	Word Frequency
Additive	3051	Food and Drug	4767	Safety Accidents	1405
Safety Accidents	2400	Safety Accidents	2632	Food and Drug	845
Canteen	1495	Additive	1228	Canteen	654
Food and Drug	1297	Highlights of food safety work arrangements	918	Education Bureau	308
Physical Health	914	Life Safety	705	Meals	255
Healthcare Food	805	Food Poisoning	701	Source	255
Safety Hazards	756	Safety Hazards	689	Life Safety	253
Aquatic Products	689	Aquatic Products	669	Foodborne	251
Life Safety	634	Source	645	National Food Safety Standards	245
counterfeit and shoddy goods	632	Healthcare Food	582	prewarning	221
Clenbuterol	622	Infant	553	Food Market	202

**Table 3:** Hot words with 6 Topics for LDA.

Topics	Topic 1	Topic 2	Topic 3	Topic 4	Topic 5	Topic 6
	Food Additives	Source Tracing	Regulation and Early Warning	Food Logistics	Campus Assurance	Upper-Level Supervision
8 words with a high frequency of occurrence	Additive	Source	Safety Accident	Diet	School Canteen	Health Bureau
	Health Food	Place of origin	Life Safety	Chain Stores	Students	Department of Health
	Raw Materials	Grain	The Law	Delivery	Education Bureau	Foodborne
	Agricultural Products	Cold Chain	Early Warning	Ordering	Kindergarten	Infectious Diseases
	Dairy Product	Agricultural Products	Security Events	Disinfection	Schools	Product Quality
	Quarantine	Food Market	Publicity and Education	Restaurants	Dining	Ministry of Health
	Poultry	Grain Bureau	Food and Drug	Selection	Raw materials	Quality Control
	Recipes	Wholesale Market	Quality	Catering Utensils	Food Poisoning	Quarantine Bureau

**Table 4:** The keywords and percentages in the categories of cluster analysis.

Category	Keywords	Percentage (%)
Supervision and Rights Protection	Department of Health; Supervisor; Sanitary Authority; Supervision Institutions; Food Poisoning; Security Control; Rights and Interests; Food Industry	8.59
Market Regulation	Counterfeit and Inferior; Security Incident; Market Supply; Disinfection; Quarantine; Quality Grain; Epidemic; Cold Chain	73.91
Catering License	Catering; Ordering; Vendors; Business license School Canteen; Quality Supervision; Physical Health; Early Warning	11.14
Food Safety Products	Food and Drug; Product Safety; Pilot work; Diet; Quality and Technical Supervision Bureau; Publicity and Education; Pharmaceuticals	6.36

**Table 5:** Statistics on the number and proportion of various policies.

First Dimension	Secondary Dimension	Number of Policies	Single Percentage %	Subtotal of Various Policies	Total Percentage %
mandatory-type	Administrative Permits	7	1.23	192	33.68
	Supervision and Sampling	45	7.89		
	Inspection and Quarantine	16	2.81		
	Law Enforcement Investigation	18	3.15		
	Regulatory System	66	11.58		
	Special Rectification	40	7.02		
capability-type	Personnel Team Construction	10	1.75	207	36.32
	Establishment and Implementation of Standards	92	16.14		
	Construction of Grassroots Institutions and Testing Institutions	10	1.75		
	Territory Management and Assessment	20	3.51		
	Emergency Plan	28	4.91		
	Signal the Potential Risks	27	4.74		
	Construction of Traceability System	4	0.71		
	Informatization Construction	16	2.81		
value-type	Create a Demonstration	16	2.81	61	10.70
	Propaganda and Guidance	31	5.44		
	Technological Innovation	14	2.46		
awards and punishments-type	Give Recognition	7	1.23	12	2.11
	Punishment Disposal	5	0.88		
innovation-type	Construction of Expert Team	19	3.33	98	17.19
	Joint Supervision	30	5.26		
	Credit Management	3	0.53		
	Openly Soliciting or Giving Opinions	46	8.07		
Total		570			100

**Table 6:** Statistics of release departments of central policies.

Department	Number of Published Policies	Proportion %
NMPA	198	34.74
Ministry of Health	72	12.63
National Health and Family Planning Commission	52	9.12
The Food Safety Commission of the State Council	37	6.49
AQSIQ	36	6.32
State Administration for Market Regulation	36	6.32
The State Administration for Industry and Commerce	28	4.91
Ministry of Commerce	23	4.04
National Health Commission	20	3.51
Certification and Accreditation Administration	10	1.75
Ministry of Education	8	1.40
Others	50	8.77
Total	570	100

## Data Availability

The data presented in this study are available on request from the corresponding author.
